# Specificity and functionality of microRNA inhibitors

**DOI:** 10.1186/1758-907X-1-10

**Published:** 2010-04-01

**Authors:** Barbara Robertson, Andrew B Dalby, Jon Karpilow, Anastasia Khvorova, Devin Leake, Annaleen Vermeulen

**Affiliations:** 1 Dharmacon Products, Thermo Fisher Scientific, 2650 Crescent Drive, Suite 100 Lafayette, CO 80026, USA; 2 Department of Chemistry and Biochemistry, University of Colorado at Boulder, UCB 215, Boulder, CO 80309, USA; 3 RXi Pharmaceuticals, 60 Prescott Street, Worcester, MA 01605, USA

## Abstract

**Background:**

Micro(mi)RNAs regulate gene expression through translational attenuation and messenger (m)RNA degradation, and are associated with differentiation, homeostasis and disease. Natural miRNA target recognition is determined primarily by perfect complementarity in a seed region (nucleotide positions 2 to 7) with additional interactions contributing in a sequence- and target-specific manner. Synthetic miRNA target analogs, which are fully complementary, chemically modified oligonucleotides, have been used successfully to inhibit miRNA function.

**Results:**

In this paper, we present a first systematic study to evaluate the effect of mismatches in the target site on synthetic inhibitor activity. Panels of miRNA inhibitors containing two-nucleotide mismatches across the target site were tested against three miRNAs (miR-21, miR-22 and miR-122). The results showed that the function of inhibitors vary as mismatch positions in the inhibitors change.

**Conclusions:**

The data indicate that features important for natural miRNA target recognition (such as seed region complementarity) are also important for inhibitor functionality. In addition, base pairing at a second, more 3' region appears to be equally important in determining the efficacy of synthetic inhibitors. Considering the importance of these inhibitor regions and the expression of closely related miRNA sequences will enable researchers to interpret results more accurately in future experiments.

## Background

Micro (mi)RNAs are small (17 to 27 nucleotides), non-coding RNAs that act in association with Argonaute (Ago) proteins to modulate gene expression via an effector nucleic acid-protein complex (microribonucleoprotein (RNP) or miRNA-induced silencing complex (RISC)). In animals, miRNA-based gene modulation occurs predominantly by the mature miRNA binding to an mRNA target site through partial base pairing, resulting in translational attenuation (for recent reviews, see [1-6]). Computational and experimental techniques for identifying target sites [7-10] have found that complementarity to the seed region (nucleotide positions 2 to 7 or 2 to 8 of the mature miRNA) is often an important determinant of target sites. In some cases of incomplete seed-pairing, pairing at '3'-compensatory' sites of the mature miRNA creates a functional target site [11,12]. The large number of potential target sites per miRNA, combined with the hundreds of putative miRNAs, has led to the prediction that a large fraction of human genes could be modulated by miRNAs.

The functional roles of miRNAs can be investigated using inhibitors, which are nucleic acid-based molecules that suppress miRNA function. Synthetic miRNA inhibitor designs incorporate the reverse complement of the mature miRNA (the target site) and are chemically modified to prevent RISC-induced cleavage, enhance binding affinity and provide resistance to nucleolytic degradation (for review see [13]). When delivered to a cell, binding of endogenous mature miRNAs to these complementary synthetic target sites is thought to be irreversible, thus these inhibitors are presumed to sequester the endogenous miRNA, making it unavailable for normal function [14-19].

To correctly associate outcomes of inhibitor experiments with specific miRNAs, it is important to understand the degree to which an inhibitor designed against one miRNA affects other miRNAs. We used synthetic inhibitors and luciferase reporters targeted by individual miRNAs to study inhibitor specificity among both natural miRNA variants in a multi-member family (let-7) and artificially designed inhibitor variants to single miRNAs (miR-21, -22, -122). Strong inhibitor crossreactivity between members of the human let-7 family, which share extensive sequence identity, was observed. Inhibitors to three different human miRNAs (miR-21, miR-22 and miR-122) were systematically mismatched at all positions, and two regions that affect inhibitor specificity were identified: the seed region (positions 3 to 8) and an additional 3' region (positions 13 to 18). These results will aid in interpretation of synthetic miRNA inhibitor studies and improvement of experimental design.

## Results

### Inhibitors of let-7 family members exhibit crossreactivity

**Figure 1:**
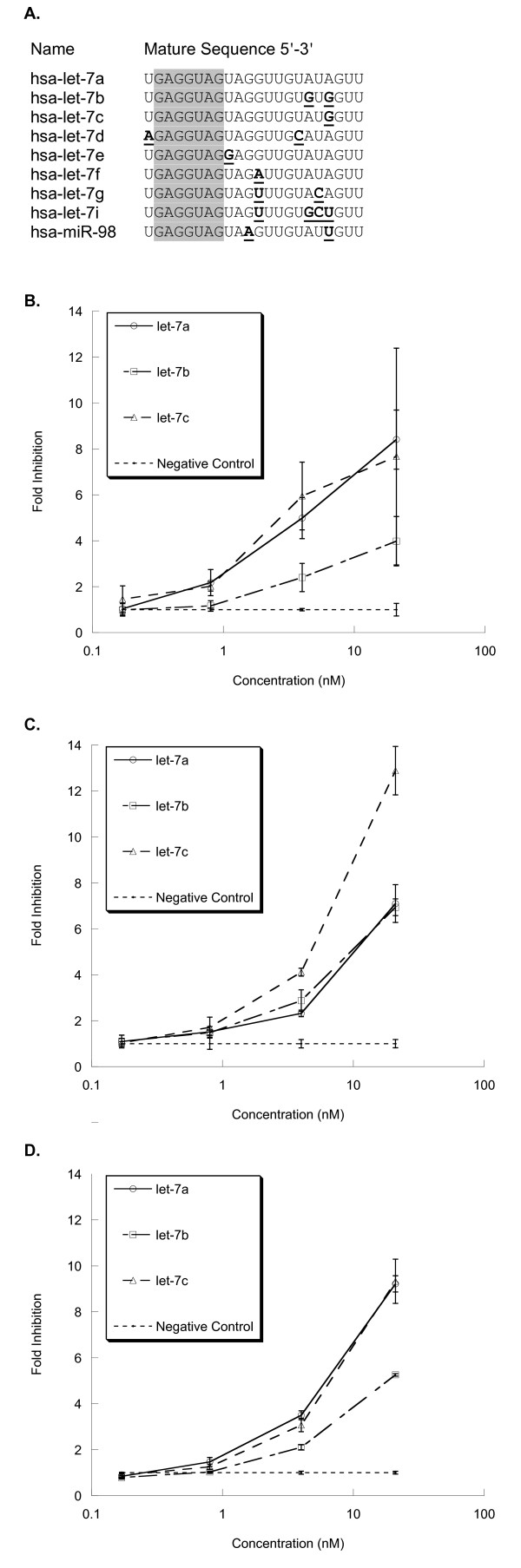
**Crossreactivity is evident between let-7 microRNA family inhibitors**. **(a) **Sequences from miRBase http://www.mirbase.org/ of the nine let-7 family members studied in this experiment. The 'seed' region (nucleotides 2 to 8), is indicated by shading. Nucleotides at which other family members differ from let-7a are underlined and in bold. **(b) **The let-7a dual-luciferase reporter was co-transfected with the negative control (NC, an equal concentration of non-functional nucleic acid molecule) or inhibitors targeting let-7a, let-7b or let-7c. **(c) **let-7b dual-luciferase reporter was co-transfected with NC or inhibitors targeting let-7a, let-7b or let-7 c. **(d) **A let-7c dual-luciferase reporter was co-transfected with NC or inhibitors targeting let-7a, let-7b or let-7c. HeLa cells were co-transfected with reporters and inhibitors 1 day after plating into 96-well plates, 10,000 cells/well, in antibiotic-free media. Inhibitor concentrations ranged from 0.17 to 21 nM; plasmid concentrations were constant at 100 ng/well. Dual-luciferase ratios were measured 2 days post-transfection. Results shown are averages from triplicate wells, normalized to appropriate controls, then expressed as fold-inhibition relative to negative control. Error bars are ± 1SD (sample) of the original triplicate data, scaled for all subsequent calculations.

To gain insight into the level of inhibitor crossreactivity to be expected between closely related family members, hairpin inhibitors (see Methods) designed against each of the nine human let-7 miRNAs (Figure 1a) were chosen for study. Some human let-7 miRNAs are expressed in many common immortal cell lines. The nine family members have sequences that differ from the canonical let-7a at either single or multiple nucleotide positions (Figure 1a). The assay system used was a set of dual-luciferase reporters for each of the let-7 miRNAs, as this type of reporter has demonstrated sufficient sensitivity to distinguish between inhibitors with only slight differences in functionality [20]. The target sites in these reporters are perfectly complementary to the mature miRNAs, because mismatched/attenuation type target sites were found to be much less sensitive [20]. All possible inhibitor/reporter pairs were tested by co-transfection into HeLa cells. The results clearly demonstrated that human let-7 miRNA inhibitors and reporter constructs, either alone or in combination, are non-specific (Figure 1b-d; also see Additional file 1, Figure S1). For each reporter, all inhibitors at 20 nM caused detectable fold changes in luciferase signal relative to the negative control. However, there was no consensus on crossreactivity ranking. For example, in both the let-7a and let-7c reporter assays, the let-7a and let-7c inhibitors caused a similar response in luciferase signal (approximately ninefold increase at 20 nM), whereas the let-7b inhibitor caused a lower response (approximately five-fold increase at 20 nM) (Figure 1b, Figure 1d). These data imply that the let-7a and let-7c inhibitors crossreact equally with each other, whereas the let-7b inhibitor has lower crossreactivity. However, in the let-7b reporter assay, the effects of the let-7a and let-7b inhibitors were similar (approximately sevenfold increase at 20 nM), whereas the effects of the let-7c inhibitor were much greater (approximately 13-fold increase at 20 nM) (Figure 1c). These latter data suggest that there is equivalent crossreactivity between the let-7a and the let-7b inhibitors, and leaves the let-7c crossreactivity open to interpretation. One plausible explanation is that multiple let-7 miRNAs are present in HeLa cells at varying concentrations and that both inhibitors and luciferase reporters crossreact with these miRNAs. The data also suggest that reporters (expressed mRNAs) and inhibitors (synthetic modified oligonucleotides) have different criteria for crossreactivity with endogenous miRNAs. Although it is possible to specifically detect expression of mature miRNAs [21-24], quantifying the level of functionally active mature miRNA (to measure inhibitor function) for closely related sequences is technically challenging. Owing to the difficulties inherent in working with multiple, related, endogenously expressed miRNAs, we decided to develop an assay in a less complex system.

### Inhibitors of non-family members do not exhibit crossreactivity

**Figure 2:**
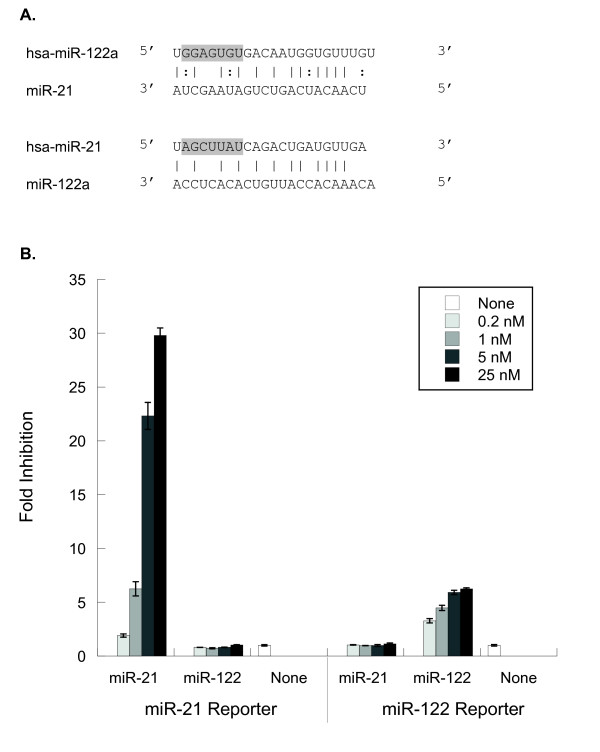
**Sequence similarity between miR-21 and miR-122 is not sufficient to cause crossreactivity between inhibitors**. **(a) **Diagram of possible pairing between **(i) **miR-122 mature (hsa-miR-122) and the miR-21 inhibitor target site, and **(ii) **miR-21 mature (hsa-mir-21) and the miR-122 inhibitor target site. The nucleotides in the seed region of the miRNA are indicated by shading. Solid lines represent Watson-Crick base pairs, and dotted lines represent G-U wobble base pairs. **(b) **Inhibitors targeting miR-21 and miR-122 were co-transfected with miR-21 or miR-122 reporters into Huh-7 cells 1 day after plating into 96-well plates, 10,000 cells/well, in antibiotic-free media. Inhibitor concentrations ranged from 0.2 to 25 nM, plasmid concentrations were constant at 100 ng/well. Dual-luciferase ratios were measured 2 days post-transfection. Results shown are averages from triplicate wells, normalized to appropriate controls, then expressed as fold-inhibition relative to no-inhibitor treatment. Error bars are ± 1SD (sample) of the original triplicate data, scaled for all subsequent calculations.

To determine crossreactivity between unrelated (non-family member) miRNA inhibitors, we focused our studies on miR-21 and miR-122. Both miRNAs are the sole human representatives of their respective families, but were found to have about 52% sequence similarity (Figure 2a). To test the level of crossreactivity, inhibitor/reporter pairs of miR-21 and miR-122 were co-transfected into Huh-7 cells, in which both miRNAs are expressed. Whereas the miR-21 inhibitor showed strong, dose-dependent inhibition of the endogenous miRNA targeting the miR-21 reporter, the miR-122 inhibitor induced no response in miR-21 reporter activity (Figure 2b). Similarly, the miR-122 inhibitor showed dose-dependent inhibition of the endogenous miRNA targeting the miR-122 reporter, whereas the miR-21 inhibitor induced no response in miR-122 reporter activity (Figure 2b). Differences in the relative amounts of endogenous miR-21 vs. miR-122 are suggested by the 30-fold vs. sevenfold respective inhibition seen at the highest inhibitor doses. Thus, no evidence of crossreactivity was detected between the miRNAs, inhibitors and reporters of the two unrelated miRNAs that share sequence similarity (Figure 2b).

### The effects of mismatches on function of inhibitors of miR-21, miR-22 and miR-122, as detected by luciferase reporters, indicate that similar regions are important in all three cases

**Figure 3:**
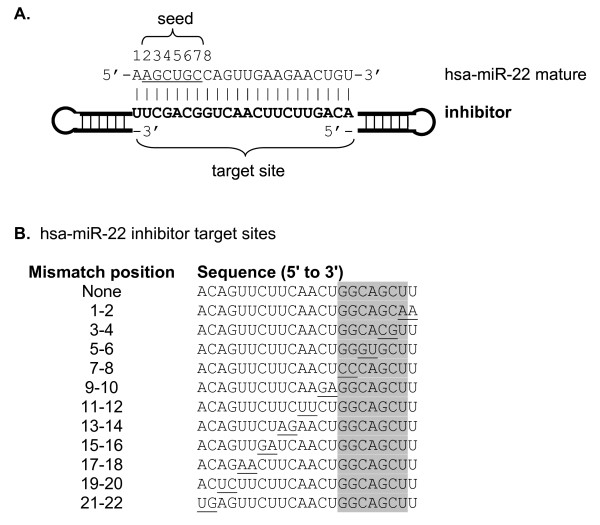
**Mismatched hairpin inhibitor design to test sequence dependence of specificity**. **(a) **Schematic showing miR-22 mature micro(mi)RNA sequence that is perfectly complementary to the central region of the hairpin inhibitor target site. **(b) **Sequences of mir-22 inhibitor target sites used in the mismatch study. Each inhibitor contained two consecutive mismatches (underlined) that are moved across the target site. Nucleotides were mismatched by substituting the reverse complement for the original nucleotide. Numbering of mismatches is from the 5' to the 3' end of the mature miRNA to which they would pair. The nucleotides that would pair with the seed region of the miRNA are indicated by shading.

In the studies described above, we found that inhibitors for miRNAs that differ at only a few nucleotides crossreacted, whereas miRNAs that differ at multiple (7 to 10) nucleotides did not. To study the effect of mismatches in inhibitor target sites (the site where the miRNA binds) on inhibitor functionality, systematically mismatched inhibitors were tested for miRNAs represented by only a single family member (for ease of data interpretation) in cell lines in which they are strongly expressed (for best signal to noise ratio in inhibitor assays) (Figure 2b; also see Additional file 1, Figure S2). For the three selected miRNAs (miR-21, miR-122 and miR-22), sets of inhibitor molecules containing two consecutive mismatches across the inhibitor target site were synthesized. The positions of these mismatches were numbered according to the positions in the mature miRNA to which they will pair (for example: positions 1 and 2, 3 and 4, 5 and 6) (Figure 3). Thus, positions numbered 2 to 7 correspond to the site where the six-mer seed region of the mature miRNA would base pair.

**Figure 4:**
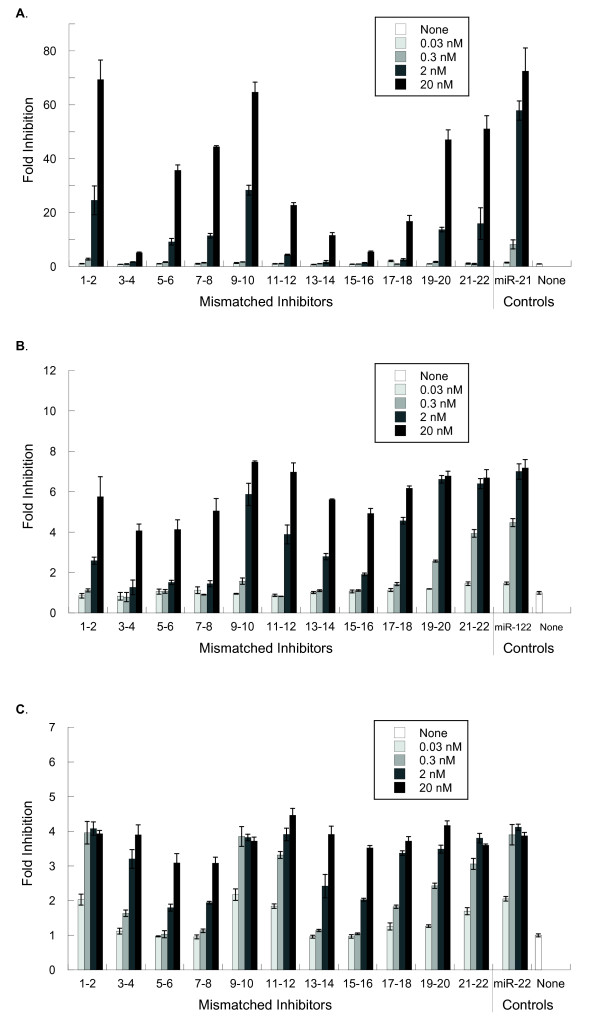
**Position of mismatches in the inhibitor target site affects inhibitor functionality**. The effect of mismatches on inhibitor function was determined for **(a) **miR-21 in HeLa cells, **(b) **for miR-122 in Huh-7 cells and **(c) **for miR-22 in HeLa cells; cell lines were chosen for high expression of the respective miRNAs. Transfections were performed 1 day after plating into 96-well plates, 10,000 cells/well, in antibiotic-free media. For each microRNA, the appropriate dual-luciferase reporter, at 100 ng/well, was co-transfected with either a fully matched inhibitor or one of a set of 11 mismatched inhibitors at concentrations from 0.03 to 20 nM. Dual-luciferase ratios were measured 2 days post-transfection. Results shown are averages from triplicate wells, normalized to appropriate controls, then expressed as fold-inhibition relative to transfection with a negative control. Error bars are ± 1SD (sample) of the original triplicate data, scaled for all subsequent calculations.

For all three miRNAs tested, mismatches causing greatest interference with inhibitor activity are located within positions corresponding to the seed region of the mature miRNAs (positions 3 to 8) and a second region closer to the 3' end (positions 13 to 18) (Figure 4). The purine and pyrmidine composition for the mismatches were examined to determine if mismatch identity affects the level of inhibition; no trend was observed in this set of data. However, for each of the three miRNAs, the exact positions within the two identified regions that were most deleterious for inhibitor function varied. The most detrimental mismatches within each miRNA were at positions 3 to 4 and 13 to 18 in miR-21 (Figure 4a; 20 nM), positions 3 to 8 and 15 to 16 in miR-122 (Figure 4b; 2 nM) and positions 5 to 8 and 13 to 16 in mIR-22 (Figure 4c; 0.3 nM). For all three miRNAs tested, mismatches located at the beginning (positions 1 and 2), end (positions 19 to 22) and middle (positions 9 and 10) of the inhibitor target site had the least effect on inhibitor function (Figure 4).

In this study, it was observed that changes in inhibitor efficacy due to mismatches were affected by miRNA expression levels and inhibitor concentration. Endogenous miRNA downregulation of the luciferase reporter, compared with the psiCHECK-2 vector without insert, suggests that miR-21, miR-22 and miR-122 are expressed at varying levels (Additional file 1, Figure S2). The effects of mismatches on inhibitor functionality were most pronounced in the case of miR-21 in HeLa cells, where approximately 70-fold inhibition was observed at the 20 nM dose for the most effective inhibitors, whereas the least effective inhibitors showed only a fivefold inhibition. However, at the 2 nM inhibitor dose, where the fully matched miR-21 inhibitor maintained approximately 60-fold inhibition, even the most effective inhibitors containing mismatches showed only 20 to 30-fold inhibition, and the least effective inhibitors containing mismatches showed no inhibition (Figure 4a). For miR-122, the best inhibitors produced approximately sevenfold inhibition at both 20 nM and 2 nM, indicating that there was a much lower amount of active endogenous miRNA present. The effects of mismatches on inhibitor efficacy were most apparent at the 2 nM dose, where the least functional inhibitors containing mismatches showed approximately 1.5-fold inhibition (Figure 4b). For miR-22, for which the maximum inhibition observed was approximately fourfold, the effects of mismatches on inhibitor efficacy were most evident at the 0.3 nM inhibitor dose (Figure 4c). Our data indicate that increased inhibitor concentration can overcome some reduction in functionality due to moderately unfavorable mismatches and, conversely, decreased inhibitor concentration can improve the specificity of inhibitors.

### The effects of mismatches on miR-122 inhibitor function, as detected by mRNA levels of an endogenous target gene, are similar to the effects observed with luciferase reporters

**Figure 5:**
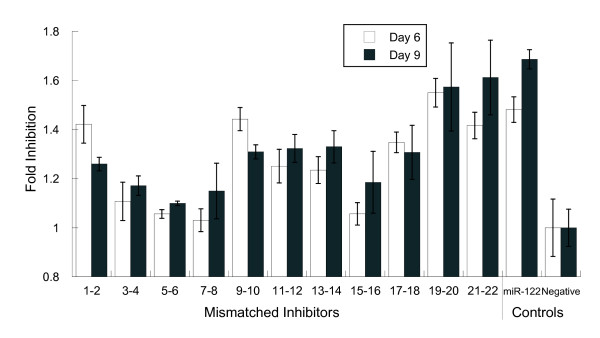
**Mismatched inhibitors show similar positional effects on the expression of an endogenous target**. The effect of mismatches on miR-122 inhibitor function was determined by measuring steady-state levels of endogenous ALDOA mRNA. The set of miR-122 inhibitors [11 mismatched and one fully matched, and a negative control (NC) inhibitor] were transfected into Huh-7 cells 1 day after plating into 96-well plates, 10,000 cells/well, in antibiotic-free media. Media was changed to new, antibiotic-free media approximately every 3 days. Response of ALDOA mRNA levels relative to a housekeeping gene was measured on day 6 (white bars) and day 9 (grey bars) post-transfection by branched-DNA assay. Results shown are averages from triplicate wells transfected with 4 nM inhibitor, normalized to appropriate controls, then expressed as fold-inhibition relative to transfection with the negative control. Error bars are ± 1SD (sample) of the original triplicate data, scaled for all subsequent calculations.

To determine whether the data observed in luciferase reporter assays could be extended to endogenous gene targets, we tested the effects of miR-122 inhibitors containing mismatches on the endogenous miR-122 target ALDOA [16-19,25]. The miR-122 mismatched inhibitors used in the reporter assays above were introduced into Huh-7 cells (doses ranging from 0.8 to 100 nM, data not shown) and mRNA levels of ALDOA were monitored. Differential responses between mismatched inhibitors were most evident for the measurements at 4 nM on day 6 (Figure 5). The mismatches producing the greatest decreases in inhibitor activity on endogenous mRNA levels (Figure 5, day 6) were those at positions 3 to 8 and 15 to 16, whereas mismatches at positions 1 to 2, 9 to 10 and 19 to 22 had the least effect on inhibitor activity compared with the fully matched inhibitor. A similar pattern was observed with the miR-122 luciferase assay (Figure 4b, 2 nM dose) suggesting that conclusions about inhibitor specificity based on reporter assays can be applied to inhibitor effects on endogenous targets.

## Discussion

### The results of this study are similar to those of other inhibitor specificity studies

Although few systematic studies of miRNA inhibitor specificity have been reported in the literature, the results from the current study are similar to those from a recent report of synthetic inhibitors in an *in vivo *animal model. Krutzfeldt *et al*. [19] tested five mismatched molecules of a synthetic miR-122 antagomir inhibitor. Consistent with our findings, (adjusting for strand numbering differences) position 5 was found to be important for function whereas mismatches to the terminal 3' position had no effect (position 23 (Krutzfeldt) and positions 21 and 22, this study). By contrast, in the current study, mismatches at positions 13 and 14 exhibited a noticeable effect on inhibitor functionality whereas Krutzfeldt *et al*. saw no effect with a mismatch at position 13 (position 14 was untested). The differences observed for position 13 relative to function of the inhibitors are probably due to the nature of the modification pattern used (two mismatches versus one mismatch) or to the sensitivity of the assay system employed. That is, subtle changes are more readily observed in cell culture with a synthetic reporter than in a mouse model documenting steady-state levels of miR-122 (by northern blotting) and target gene mRNA (by reverse transcriptase PCR) in liver preparations.

### Small differences in mismatched inhibitor efficacy between the three miRNAs are probably due to sequence specific effects

Although regions of importance common to all three miRNA inhibitors tested were very evident, there were slight differences observed in inhibitor responses to mismatches at particular locations. For example, within the seed region, the 3 to 4 mismatch was especially detrimental for miR-21 inhibitor function, whereas it was the least perturbing for miR-22 inhibitor function. One reasonable explanation for such differences may be sequence dependence, but in the example given, the mismatched pair was CG for both miR-21 and miR-22 (see Additional file 1, Table 2). Although we could discern no obvious sequence-dependent pattern in our limited set of comparisons, several studies performed to assess the specificity of binding between a small interfering (si)RNA or miRNA and the target mRNA have shown that the identity of the specific nucleotide(s) can influence the effect of a mismatch at a particular site [11,26-28]. While the miRNA-synthetic inhibitor interaction is analogous to the miRNA-mRNA interaction, we acknowledge that inhibitor 2'-*O*-methyl nucleotide modifications increase binding affinity to the mature miRNA and this limits comparison of our study with the miRNA/siRNA-mRNA studies.

### Comparisons between miRNA-inhibitor effects and miRNA-mRNA interactions

In our study, we found positions 3 to 8 in the inhibitor to be important for miRNA inhibition. These data are in agreement with many other studies, which have found the seed region to be a determinant of miRNA-mRNA target recognition [9]. Therefore, seed binding during target recognition is a common feature, despite the difference between the experimentally induced miRNA-inhibitor interaction (RNA-synthetic 2'-*O*-methyl-modified oligonucleotide) that produces irreversible sequestration of the miRNA and the endogenous miRNA-mRNA (RNA-RNA) interaction that results in gene modulation.

The 3' region, positions 13 to 18, found in the current study to be crucial for miRNA-inhibitor recognition, roughly corresponds to the '3' compensatory' or 'beneficial 3' pairing' sites identified in miRNA-mRNA recognition of target sites in expressed reporter 3' untranslated regions [11,12]. However, in contrast to effects described previously, we found that with synthetic inhibitors, the 13 to 18 positional effects were as strong as the effects observed for positions 3 to 8. The variable magnitudes of effect are presumably related to differences in the type of target binding that is occurring in the two types of studies. Recent elucidation of an Argonaute protein-DNA guide strand-RNA target ternary complex crystal structure supports a 'two-state' model for target binding [29]. This is often described as a nucleation step initiating binding at the seed followed by 'zippering' to form the remainder of the duplex [30]. It seems reasonable to propose that the chemical modification of the inhibitors might strongly affect this 'zippering' process.

Analogous to the interaction between mature miRNA and inhibitor, *in vitro *studies of miRNA duplex unwinding in *Drosophila melanogaster *[31] and humans [32] have found similar positional effects, with mismatches in the seed and a 3' region having strong effects on unwinding of the mature and passenger miRNA strands. The authors concluded that the importance of mismatches in the seed and the 12 to 15 nucleotide regions for unwinding is the reverse of the requirements for complete base pairing in those regions for miRNA-mRNA recognition.

### Results of this study help predict which inhibitors may crossreact

The mismatch data presented in this study offer guidelines for predicting when synthetic, 2'-*O*-methyl miRNA inhibitors may crossreact. Mature miRNA family members that share sequence identity across both the seed (nucleotides 2 to 8) and the 3' critical region (nucleotides 13 to 18) are likely to have inhibitors that crossreact. An example of this is the hsa-miR-15a and hsa-miR-15b pair, which differ from each other at only a few nucleotides, all of which are outside the seed and the 3' critical region. Details of nucleotide pairing for examples from the human miR-15 family (miRBase, http://www.mirbase.org/) are shown in Table 1. A contrasting example, of family members for which specific inhibitors might be designed, is the hsa-miR-15a and hsa-miR-16 pair, which share sequence identity in the seed but differ at nucleotides 14 to 16 within the 3' critical region. Mature miRNAs that are not family members may share considerable sequence identity, because family members are identified in miRBase by homology of the entire hairpin, not just the mature sequence. An example of this is the hsa-miR-15a and hsa-miR-497 pair, which share seed sequences and are also identical at five out of six positions within the 3' critical region (Table 1). Because specific sequence contributions are known to affect interactions, it is recommended to test for crossreactivity whenever there is extensive identity in both the seed and the 3' critical regions. Therefore, although it is not possible to establish absolute criteria for predicting inhibitor crossreactivity, knowledge of the regions important in determining specificity enables identification of potential crossreacting miRNAs, which can then be tested.

**Table 1:** Examples showing alignment of mature sequences from miR-15 family members and non-family member with matching seeds

Family member	miRNA name	Mature sequence from 5' to 3' end
Family members with sequence identity in both the seed region^a ^and the 3' region^b^	hsa-miR-15a^c^	U***AGCAGCA***CAUA**AUGGUU**UGUG
		| | | | | | | | | | | | | | | | | |
	hsa-miR-15b^c^	U***AGCAGCA***CAUC**AUGGUU**UACA
Family members with sequence identity in the seed region^a ^but very limited identity in the 3' region^b^	hsa-miR-15a^c^	U***AGCAGCA***CAUA**AUGGUU**UGUG
		| | | | | | | | | | | | | | | |
	hsa-miR-16^c^	U***AGCAGCA***CGUA**AAUAUU**GGCG
Non-family members with sequence identity both in the seed region^a ^and in the 3' region^b^	hsa-miR-15a^c^	U***AGCAGCA***CAUA**AUGGUU**UGUG
		| | | | | | | | | | | | | | | | |
	hsa-miR-497^d^	C***AGCAGCA***CACU**GUGGUU**UGU

^a^Bold and italicised.

^b^Bold.

^c^Member of the miR-15 family.

^d^Member of the miR-497 family.

### Crossreactivity of inhibitors could be an advantage in some cases

miRNA family members that are sufficiently similar to exhibit inhibitor crossreactivity might also reasonably be expected to have redundant biological function if co-expressed [33,34]. Therefore, inhibiting multiple family members at one time could reveal a loss of function phenotype that would be difficult to observe by traditional genetic knockout studies [35,36]. Owing to the high potency of the studied synthetic inhibitors relative to endogenous levels of all but the most strongly expressed miRNA, most inhibitors could be delivered at low doses and still be highly effective [20]. In addition, due to the loss of inhibitor specificity observed at higher doses, use of the lowest effective dose is a desirable goal. In this context, in cases where miRNAs of very similar sequence are known, an experimental approach likely to produce consistent results would be the use of a pool of inhibitors designed against a group of related miRNAs, each at a low dose, rather than a single inhibitor.

## Conclusions

We have conducted a systematic study of the effect on inhibitor functionality of mismatches in the target sites of three human miRNA inhibitors. We conclude that the seed region (position 3 to 8) and a 3' region (position 13 to 18) are equally important in determining recognition of the inhibitor target by endogenous miRNAs for the type of chemically modified, synthetic inhibitor we studied. From the literature, it is apparent that these rules may not be universally applicable to other types of inhibitors, especially those involving multiple, endogenously expressed target sites. For our design of inhibitor, we can use results of the mismatch study to identify potential crossreacting miRNAs in the miRBase database. A recommendation that arises from these results is that use of a pool of inhibitors targeting all of the potential crossreacting miRNAs should produce the most consistent results from inhibitor experiments. Owing to the strong influence of dose on inhibitor specificity, use of the minimal effective dose is recommended.

## Methods

### miRNA inhibitors

All inhibitors were fully 2'-*O*-methylated molecules (miRIDIAN Hairpin inhibitor design; Dharmacon Products, Thermo Fisher Scientific, Lafayette, CO, USA) [20] (Figure 3a). The negative control inhibitors used in these studies are based on *Caenorhabditis elegans *miRNAs not found in humans [miRIDIAN miRNA Hairpin Inhibitor Negative Controls 1 (cat. no. IN-001005-01) and 2 (cat. no. IN-002005-01)]. Mismatched inhibitors contain two consecutive mismatches (for example, positions 1 and 2, 3 and 4, 5 and 6) across the target site. Sequences for all inhibitor target sites in this study are reported in Figure 3 or Additional file 1, Table S1 and Table S2.

### Cell culture and transfection

Huh-7, a liver-derived cell line from Japan (gift of T. Hodges), was cultured in modified Eagle's medium (high glucose) with 10% fetal bovine serum and 2 mM L-glutamine. This cell line is known to have moderate to high endogenous levels of miR-122 [17,25,37]. All other cell lines used in this study were obtained from the American Type Culture Collection and cultured under recommended conditions.

For transfection, cells were plated into 96-well plates, with 10,000 cells/well in serum-containing media without antibiotics, and transfected approximately 24 hours after plating. For luciferase assays, the test inhibitor molecule and the respective dual luciferase reporter or control plasmid (cat. no. C8021; psiCHECK™-2; Promega, Foster City, CA, USA) were co-transfected into cells using transfection reagents (cat. no. 2010; DharmaFECT^® ^Duo [Thermo Fisher Scientific] or cat. no. 11668; Lipofectamine 2000 [Invitrogen Corp., Carslbad, CA, USA]) according to the manufacturers' instructions, at 0.2 to 0.4 μg/well. Inhibitor concentrations varied between approximately 0.03 and 21 nM, whereas reporter and control plasmid concentrations remained constant at 100 ng/well.

For assays of the endogenous human aldolase A (ALDOA) gene (NM_000034) response to miRNA modulation, cells were transfected with the miR-122 hairpin inhibitor over a concentration range of 0.8 to 100 nM using 0.2 μg/well transfection reagent (cat. no. T-2001; DharmaFECT 1; Thermo Fisher Scientific), according to the manufacturer's instructions. In cases where cells were cultured for longer than 3 days post-transfection, the media was replaced with new growth media approximately every 3 days.

### Dual-Luciferase reporters and assay

The dual-luciferase reporters were all derived from the psiCHECK-2 vector (cat. no. C8021; Promega) and contain a single target site, fully complementary to the mature miRNA [38-40] (miRBase; http://www.mirbase.org/) cloned into the the 3' untranslated region of the modified *Renilla luciferase *(hRluc) gene. These constructs were used in conjunction with a commercial assay system (cat. no. E2920, E2940 and E2980; Dual-Glo™ Luciferase Assay System; Promega). Because this assay measures protein activity, these reporters show luciferase response from both cleavage and non-cleavage targeting [20]. Assays were performed 48 hours post-transfection; further details of assay design and instrumentation are provided in Vermeulen *et al*. [20]. *Renilla *luciferase/firefly luciferase (Rluc/Fluc) values are the average of triplicate wells; error bars are ± 1 SD (sample) of the original average, scaled for subsequent ratio calculations. Reported values are the ratio of the reporter plasmid signal to the control plasmid (psiCHECK-2) signal normalized to the ratio obtained from co-transfection with matched amounts of non-targeting inhibitor controls.

## Cell viability

Cell viability was assessed using an indicator dye (alamarBlue^®^; BioSource International, Inc., Camarillo, CA, USA) according to the manufacturer's instructions. For all data reported in this study, no cell viability differences were observed between the reporter and control (psiCHECK-2) plasmid treatments (data not shown).

### Endogenous ALDOA mRNA quantification

Levels of both ALDOA and peptidyl-prolyl *cis-trans *isomerase B (PPIB) (NM_000942, measured as the reference housekeeping gene) mRNAs were determined using the branched DNA assay (cat. no. QG-000-050; QuantiGene Screen Kit; Panomics Inc., Fremont, CA, USA) [41]. Knockdown of ALDOA was assessed as a change in the ratio of ALDOA to PPIB with different treatments, measured at 6 and 9 days post-transfection.

## Competing interests

The authors declare that they have no competing interests.

## Authors' contributions

BR conceived of and designed the specificity studies, carried out assays and analyzed results, and drafted the manuscript. AD carried out luciferase assays and analyzed the results. AK participated in design and in interpretation of results. JK participated in the design, analysis of results and drafting, and critical review of the manuscript. DL participated in the design, analysis of results and critical review of the manuscript. AV participated in design, coordination of execution and analysis of all experiments, and drafting and critical review of the manuscript. All authors read and approved the final manuscript.

## Supplementary Material

Additional file 1 **Supplemental Figures and Tables**. Supplemental Figure S1 - All let-7 family member reporter assays show crossreactivity between all let-7 inhibitors, although the degree of apparent crossreactivity varies across reporters. Supplemental Figure S2 - Dose curves of fully complementary inhibitors co-transfected with cognate reporters. Supplemental Table S1 - Sequences of let-7 inhibitor target sites. Supplemental Table S2 - Sequences of mismatched inhibitor target sites. Click here for file
