# Supplementary material for: Specificity and functionality of microRNA inhibitors
Source: Silence. 2010 Apr 1;1:10. doi: 10.1186/1758-907X-1-10 (PMC2864222; doi:10.1186/1758-907X-1-10)

Supplementary Figures and Tables

Supplemental Table 1

let-7 hairpin inhibitors

miRNA	Target site sequence (5' to 3')
let-7a	AACUAUACAACCUACUACCUCA
let-7b	AACCACACAACCUACUACCUCA
let-7c	AACCAUACAACCUACUACCUCA
let-7d	AACUAUGCAACCUACUACCUCU
let-7e	AACUAUACAACCUCCUACCUCA
let-7f	AACUAUACAAUCUACUACCUCA
miR-98	AACAAUACAACUUACUACCUCA
let-7g	AACUGUACAAACUACUACCUCA
let-7i	AACAGCACAAACUACUACCUCA

Supplemental Table 2

miRNA	Mismatch Position	Target site sequence (5' to 3')
**miR-21 hairpin inhibitors**		
miR-21	1 & 2	UCAACAUCAGUCUGAUAAGC**AU**
miR-21	3 & 4	UCAACAUCAGUCUGAUAA**CG**UA
miR-21	5 & 6	UCAACAUCAGUCUGAU**UU**GCUA
miR-21	7 & 8	UCAACAUCAGUCUG**UA**AAGCUA
miR-21	9 & 10	UCAACAUCAGUC**AC**AUAAGCUA
miR-21	11 & 12	UCAACAUCAG**AG**UGAUAAGCUA
miR-21	13 & 14	UCAACAUC**UC**UCUGAUAAGCUA
miR-21	15 & 16	UCAACA**AG**AGUCUGAUAAGCUA
miR-21	17 & 18	UCAA**GU**UCAGUCUGAUAAGCUA
miR-21	19 & 20	UC**UU**CAUCAGUCUGAUAAGCUA
miR-21	21 & 22	**AG**AACAUCAGUCUGAUAAGCUA
miR-21	matched	UCAACAUCAGUCUGAUAAGCUA
**miR-122 hairpin inhibitors**		
miR-122	1 & 2	ACAAACACCAUUGUCACACUC**GU**
miR-122	3 & 4	ACAAACACCAUUGUCACAC**AG**CA
miR-122	5 & 6	ACAAACACCAUUGUCAC**UG**UCCA
miR-122	7 & 8	ACAAACACCAUUGUC**UG**ACUCCA
miR-122	9 & 10	ACAAACACCAUUG**AG**ACACUCCA
miR-122	11 & 12	ACAAACACCAU**AC**UCACACUCCA
miR-122	13 & 14	ACAAACACC**UA**UGUCACACUCCA
miR-122	15 & 16	ACAAACA**GG**AUUGUCACACUCCA
miR-122	17 & 18	ACAAA**GU**CCAUUGUCACACUCCA
miR-122	19 & 20	ACA**UU**CACCAUUGUCACACUCCA
miR-122	21 & 22	A**GU**AACACCAUUGUCACACUCCA
miR-122	matched	ACAAACACCAUUGUCACACUCCA
**miR-22 hairpin inhibitors**		
miR-22	1 & 2	ACAGUUCUUCAACUGGCAGC**AA**
miR-22	3 & 4	ACAGUUCUUCAACUGGCA**CG**UU
miR-22	5 & 6	ACAGUUCUUCAACUGG**GU**GCUU
miR-22	7 & 8	ACAGUUCUUCAACU**CC**CAGCUU
miR-22	9 & 10	ACAGUUCUUCAA**GA**GGCAGCUU
miR-22	11 & 12	ACAGUUCUUC**UU**CUGGCAGCUU
miR-22	13 & 14	ACAGUUCU**AG**AACUGGCAGCUU
miR-22	15 & 16	ACAGUU**GA**UCAACUGGCAGCUU
miR-22	17 & 18	ACAG**AA**CUUCAACUGGCAGCUU
miR-22	19 & 20	AC**UC**UUCUUCAACUGGCAGCUU
miR-22	21 & 22	**UG**AGUUCUUCAACUGGCAGCUU
miR-22	matched	ACAGUUCUUCAACUGGCAGCUU

Supplementary Figure Legends

Supplemental Figure 1 – All let-7 family member reporter assays show cross reactivity among all let-7 inhibitors, although the degree of apparent cross-reactivity varies across reporters.

Inhibitors targeting let-7a, b, c, d, e, f, g, i or miR-98 were co-transfected with dual luciferase reporters for each family member into HeLa cells one day after plating in 96-well plates, 10,000 cells/well, in antibiotic-free media. Inhibitor concentrations ranged from 0.17 to 21 nM, plasmid concentrations were constant at 100 ng/well. Dual-luciferase ratios were measured two days post-transfection. Results shown are averages from triplicate wells, normalized to appropriate controls, then expressed as fold-inhibition relative to negative control. Error bars are +/- one sample standard deviation of the original triplicate data, scaled for all subsequent calculations.

Supplemental Figure 2 – Dose curves of fully complementary inhibitors co-transfected with cognate reporters.

The dose curves are shown normalized in two ways: (A) Average Rluc/Fluc ratio for the miRNA reporter normalized to the psiCHECK-2 controls. The levels of luciferase knockdown at no inhibition and the concentration of inhibitor required for ~ 50 % inhibition demonstrate relative expression of the miRNAs from high to low as miR-21 > miR-122 > miR-22. (B) Average Rluc/Fluc ratio that was plotted in (A) normalized to endogenous expression (no inhibition) to show fold inhibition as in the main body of the paper. The fold inhibition suggests relative expression from high to low as miR-21 >> miR-122 > miR-22. miR-21 and miR-22 were assayed in HeLa cells, miR-122 was assayed in Huh7 cells.

Supplemental Figure 1

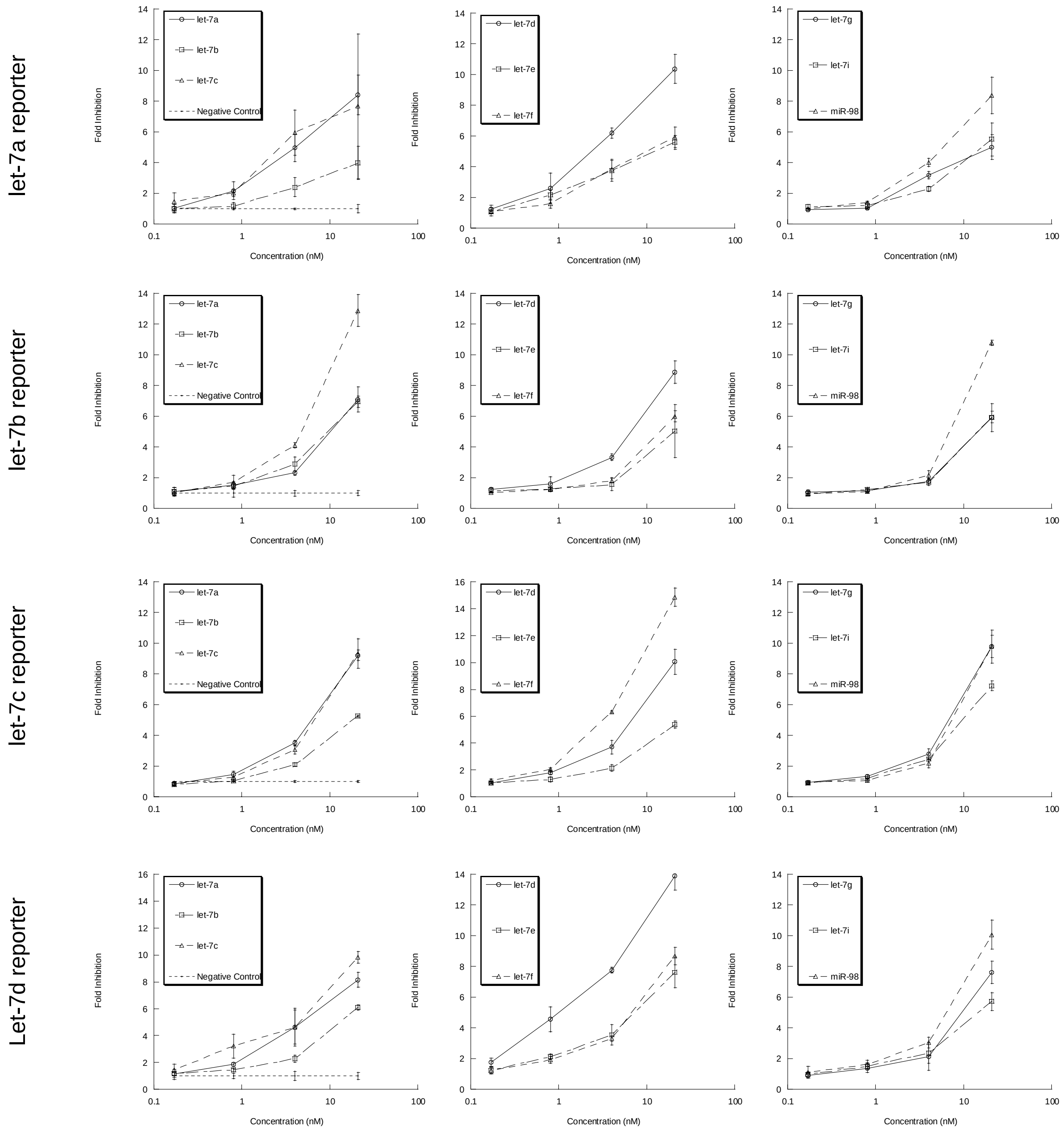

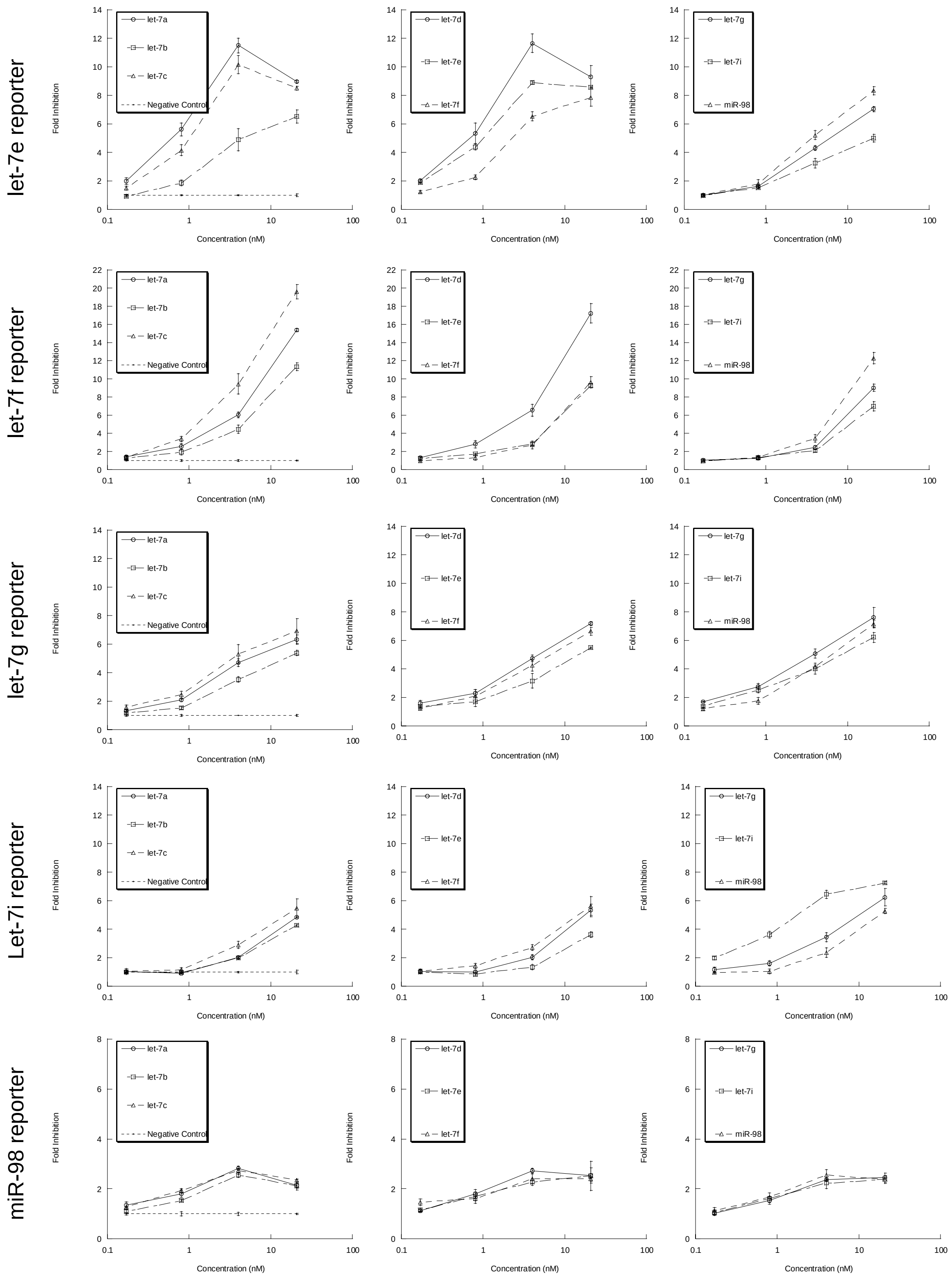
let-7e reporter
let-7f reporter
let-7g reporter
Let-7i reporter
miR-98 reporter
let-7a
let-7b
let-7c
Negative Control
let-7d
let-7e
let-7f
let-7g
let-7i
miR-98
Fold Inhibition
Concentration (nM)

Supplemental Figure 2

A.

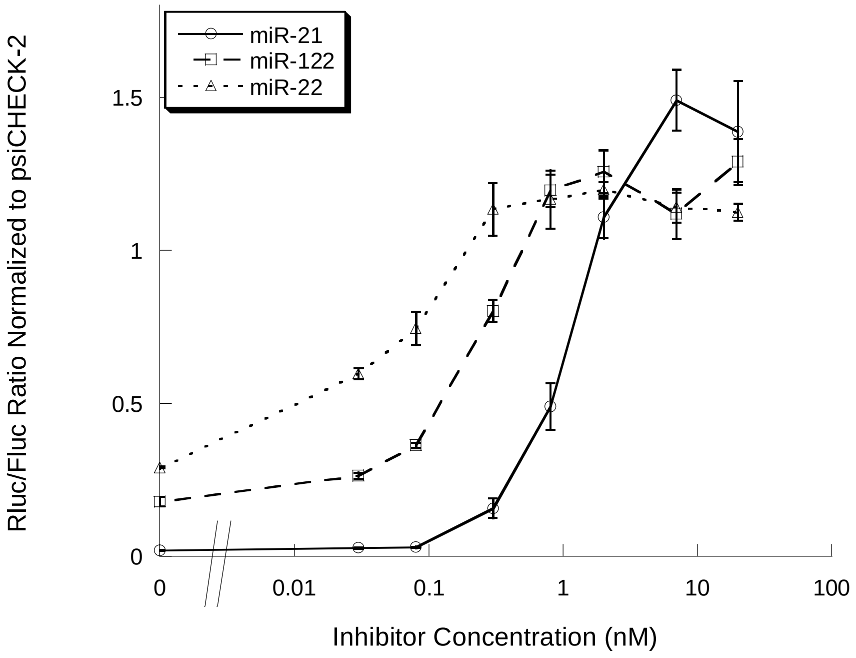

B.

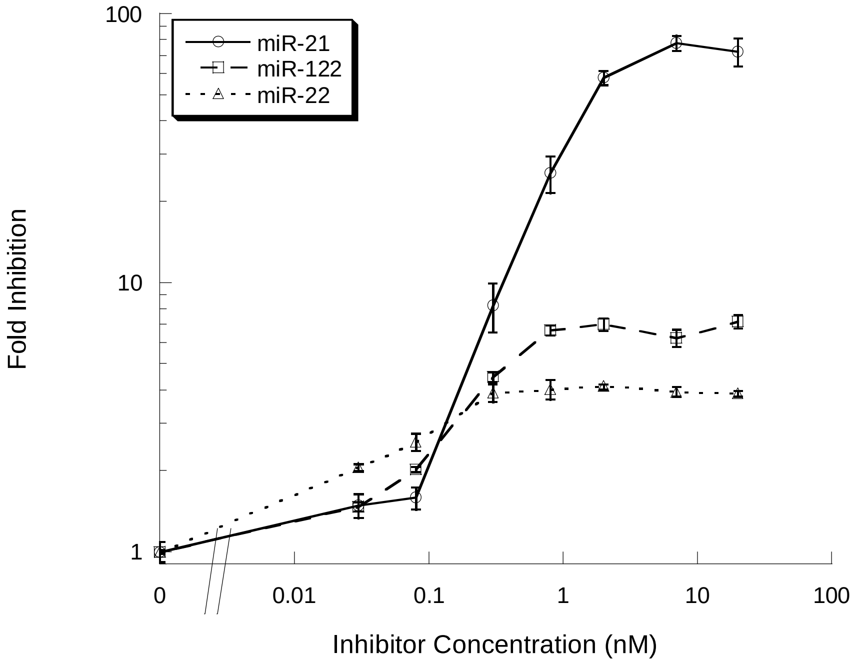

Supplement: Additional file 1 — Supplemental Figures and Tables. Supplemental Figure S1 - All let-7 family member reporter assays show crossreactivity between all let-7 inhibitors, although the degree of apparent crossreactivity varies across reporters. Supplemental Figure S2 - Dose curves of fully complementary inhibitors co-transfected with cognate reporters. Supplemental Table S1 - Sequences of let-7 inhibitor target sites. Supplemental Table S2 - Sequences of mismatched inhibitor target sites. [file 1758-907X-1-10-S1.PDF]
